# Cosmetic operative care abroad leads to a multidrug-resistant *Mycobacterium abscessus* infection in a patient: a case report

**DOI:** 10.1186/s13256-022-03678-z

**Published:** 2022-11-29

**Authors:** Karam R. Motawea, Randa K. Rabea, Rowan H. Elhalag, Jason Goodloe, Dina M. Awad, Manmeet Kaur, Ahmed K. Awad, Sarya Swed, Joseph Varney

**Affiliations:** 1 Alexandria Faculty of Medicine, Alexandria University, Alexandria, Egypt; 2 Faculty of Medicine, Ain Shams University, Cairo, Egypt; 3 School of Medicine, American University of the Caribbean, Philipsburg, Sint Maarten; 4 Faculty of Medicine, Aleppo University, Aleppo, Syria

**Keywords:** *Mycobacterium abscessus*, Cosmetic surgery, Infectious disease

## Abstract

**Introduction:**

The *Mycobacterium abscessus* complex is a nontuberculous mycobacteria species that is pervasive in soil and water. Various medical equipment malfunctions, infected surfaces, and patient transmission are potential causes of *Mycobacterium abscessus* infection in the hospital environment. These cases have an annual prevalence that ranges from 1.4 to 6.6 per 100,000 infections, mainly increasing.

**Case presentation:**

We present the case of a 23-year-old American female patient who presented to the emergency room with significant abdominal pain between low pelvic sutures and the umbilicus. She reported abdominal pain, pruritus, and boils on her back preventing her from standing upright. The symptoms occurred in the liposuction area after cosmetic surgery in the Dominican Republic. The clinical, radiological, and cultural findings helped diagnose *Mycobacterium abscessus* infection. We conducted a mini literature review on the published reports of *Mycobacterium abscessus*.

**Conclusion:**

*Mycobacterium abscessus* infection may occur due to surgical procedures abroad. Measures are required to combat *Mycobacterium abscessus* and reduce its prevalence in hospital settings.

## Background

In the hospital environment, various problems, such as malfunction of the medical equipment, infected surfaces, and patients spreading various infections, are documented as potential causes of infections [1]. One such possible agent is *Mycobacterium abscessus*. This organism was first isolated in 1952 via aspiration of a knee abscess [2]. Reclassification of *M. abscessus* took place not that long ago, in 1992; it had been previously classified in the same species as *M. chelonae* [2]. *M. abscessus* complex is a class of swiftly growing, multidrug-resistant nontuberculous mycobacteria (NTM) species that is pervasive in soil and water [2].

These cases show an annual prevalence that ranges from 1.4 to 6.6 per 100,000 infections, mainly increasing. *M. abscessus* infections are relatively more common in females than males and significantly more in patients over the age of 60 years [3]. Owing to its extensive multiple drug resistances, treatment of *M. abscessus* infection requires a more refined approach [4]. Furthermore, *M. abscessus* is multidrug resistant and resistant to various disinfectant agents, deeming postsurgical and postprocedural infectious occurrences by *M. abscessus* more unfavorable than other types of bacteria [4, 5]. We present a case of *M. abscessus* in a patient who traveled to the Dominican Republic to receive cosmetic surgery, which resulted in a deep tissue infection that was later treated in the USA. A literature review of the published reports was also carried out.

## Case report

A 23-year-old American female patient with 2-week history of blistering and drainage from suture sites from recent cosmetic surgery (abdominoplasty, liposuction of abdominal flanks and back, with immediate fat transfer to the gluteal region in the Dominican Republic on 5 March 2020), presented to the emergency department (ED) with severe abdominal pain between low pelvic sutures and umbilicus, preventing her from being able to stand up straight, pruritis and boils on her back from the area of liposuction preventing her from being able to stand up straight. The patient denied fever and chills. Her blood pressure and pulse were 122/78 mmHg and 101 beats per minute, respectively. General appearance showed an overweight female with three linear open wounds along with incision sites: two on the right and one on the left with residual scars, with clear yellow drainage, erythematous and tender area, and areas of burn and dried scabs on back. The patient had no significant past medical history.

Abdominal examination showed a morbidly obese patient with a non-distended, soft, tender abdomen to palpation around the wound, multiple open draining sinuses along the transverse incisional scar, and the base of openings with healthy granulation tissue. There was no erythema, with no warmth to touch. Respiratory, cardiac, musculoskeletal, and neurological examinations were normal. The patient reported that she was being kept in a “recovery clinic” in the Dominican Republic after the cosmetic operation, and because she complained of severe discomfort from her back drain, the doctor removed the posterior drain. She eventually left the clinic and the Dominican Republic against medical advice and flew back to the USA (11 March 2020).

After arriving from the Dominican Republic, she treated her back pain with a heating pad, causing a partial-thickness burn. She was then treated at Nassau University Medical Center (NUMC) Burn Clinic for her partial-thickness burns. The patient reported that she had multiple draining sinuses along her transverse abdominal incision, as well as her umbilicus. She had been packing the sinuses by herself at home with gauze, wounds continued to drain, and she completed a course of cefalexin and doxycycline after admission to urgent care. The patient was also seen at Good Samaritan Hospital, which recommended trimethoprim–sulfametoxazole, which the patient did not take. She was treated with oxycodone–acetaminophen 10 mg, acetaminophen 1 g, and clindamycin 900 mg at the emergency department.

**Fig. 1:**
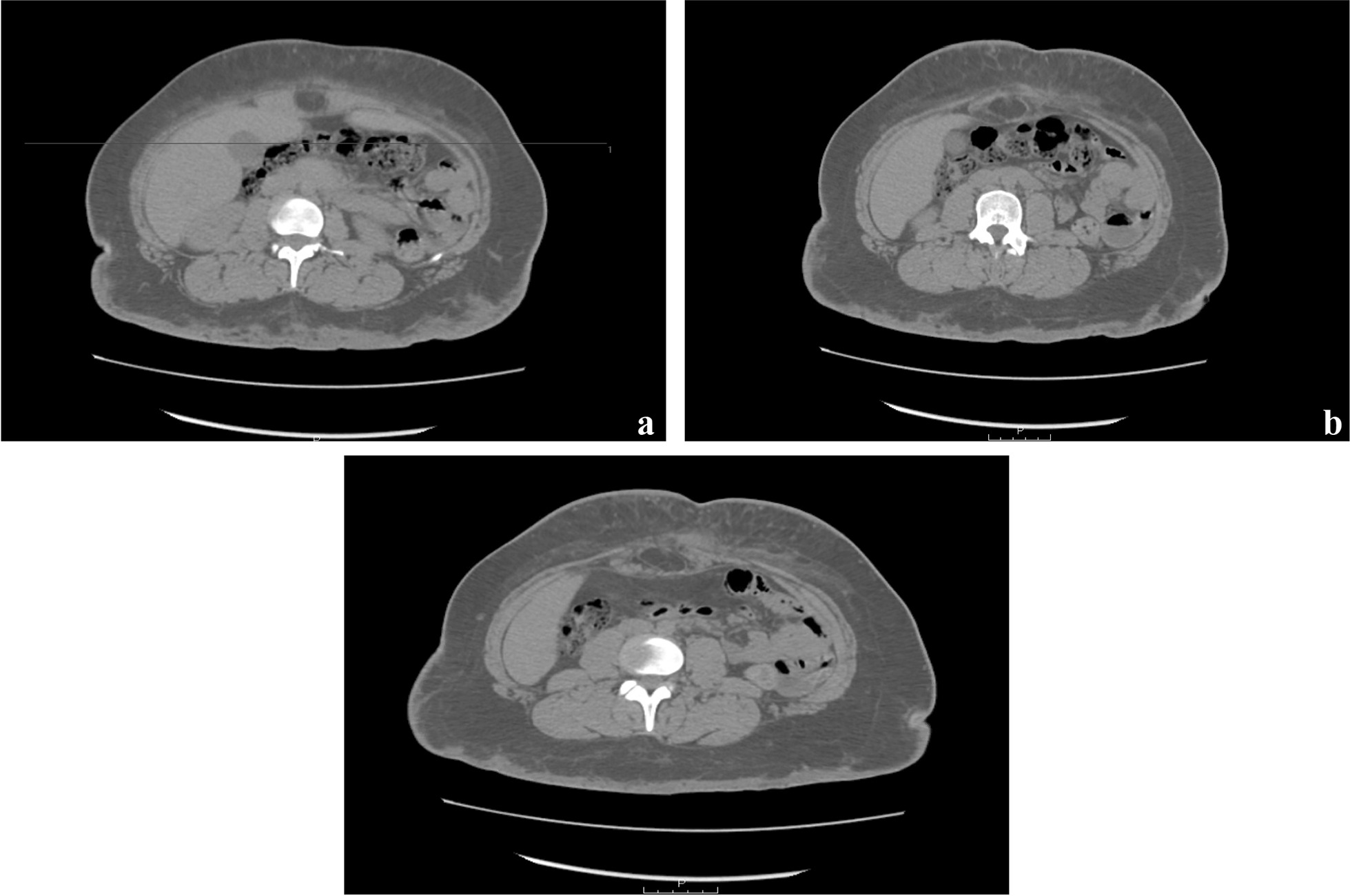
**a**–**c** Abdominal scan showing *Mycobacterium abscessus* complex

Computed tomography (CT) of abdomen and pelvis (Fig. 1) was performed and showed a minor fat-containing umbilical hernia, status post-Roux-en-Y gastric bypass, no evidence of afferent limb dilation, no evidence of obstruction, unremarkable appendix and terminal ileum, and reactive bilateral inguinal lymphadenopathy, with the largest node measuring up to approximately 1.5 cm on the left. The CT also showed infiltration of the lower abdominal soft tissues containing an 11.9 × 1.4 × 4.4 cm^3^ fluid collection compatible with abscess. Several foci of air were noted within the right side of this fluid collection, tracking to a soft tissue defect in the right pyramid line lower abdomen. There was a thickening of the skin and subcutaneous fat-containing globules in the gluteal soft tissues. The urinary bladder was collapsed. The uterus and adnexa were not enlarged.

A management plan was put in place for the patient after the CT, which included intravenous hydration, clindamycin 600 mg, pain control with oxycodone, follow-up blood cultures, interventional radiology (IR) for image-guided drainage, plastic surgery, and wound consult, and skincare referral. IR procedure was done. A fluid culture was sent to the laboratory, and the sample was also to be tested for acid-fast bacillus (AFB). Blood culture was done and showed no growth after 5 days, the abdominal fluid culture showed no growth after 48 hours, and abdominal fluid AFB showed no growth after 24 hours. After 8 days, the culture showed acid-fast bacilli in the liquid culture medium, and the DNA probe result was negative for *M. tuberculosis*.

The management plan at this stage included no clinical indication for the patient to receive antibiotics at this time; the patient was to be discharged with pain medication, proper irrigation and debridement, daily packing, sterile dressing change, and a plastic surgery appointment was scheduled within the following 2 weeks. Laboratory investigations were ordered and noted as white blood cell (WBC) count: 4.28 × 10^9^/L.

Under real-time ultrasound guidance by IR procedure, access to the fluid collection was obtained with a 19G single-wall needle. Approximately 7 cm^3^ of cloudy fluid was aspirated and sent for appropriate analysis. After 25 days, the bacterial culture showed *M. abscessus* complex on 21 June. The patient was prescribed doxycycline for 10 days and azithromycin 500 mg. On 29 June, the left side of the wound almost completely healed, with few open draining tracts on the right side and some minor erythema. The patient was advised to continue on the recommended antibiotics, continue local wound care, and come to the ED for worsening pain, erythema, and drainage from wounds.

## Discussion

Cosmetic surgery is any technique that improves the patient’s appearance by increasing symmetry, esthetic appeal, and proportion, according to the American Board of Cosmetic Surgery. This broad category includes a variety of operations carried out using chemical agents, surgery, laser therapy, and mesotherapy (hydrocolloid fillers and botulinum toxin). Physiologic hazards are comparable to lower in plastic surgery operations than in other surgical subspecialties. Typically, elective cosmetic surgical operations are carried out on reasonably healthy patient groups as outpatient procedures. Even with all of these things, postoperative problems still carry a high risk. Infections, local anesthetic systemic toxicity (LAST), anomalies of the electrolytes and hematology, intravascular fluid changes, and wound problems are common consequences. Surgical-site hematomas and LAST are examples of postoperative complications that might develop immediately or months later [1, 2]. We report a 23-year-old female patient who went to the Dominican Republic for cosmetic surgery (abdominoplasty liposuction of abdominal flanks and back, with immediate fat transfer to the gluteal region) and came back to the USA with *M. abscessus* infection. She developed symptoms 2 weeks after the procedure. A total of 20 travelers with *M. abscessus* infection were detected upon returning to the USA. In these patients, who all went to the Dominican Republic for cosmetic surgery, the doctors used the Emerging Infections Network (EIN) to classify *M. abscessus* wound infection [6]. Furuya *et al*. reported eight previously healthy Hispanic women patients who underwent abdominoplasties at the same clinic in the Dominican Republic. Symptoms in these patients first developed 2–18 weeks postprocedure [7].

*Mycobacterium abscessus* complex infection can be obtained in both the community and the hospital setting. In the community setting, water supply systems have been known to be the source of human infections [5, 7]. Given the potential for vulnerable hosts with underlying structural issues and illnesses to be the source of the tainted water supply system, *M. abscessus* infections may be more likely to develop in such hosts [5]. *Mycobacterium abscessus* complex-related pulmonary illness normally progresses slowly but steadily over time, resulting in deteriorated quality of life, chronic symptoms, and decreased lung function; however, the disease may sometimes proceed quickly and result in sudden respiratory failure [5].

*Mycobacterium abscessus* is a slow-growing bacterium found in the environment that is resistant to many antibiotics and disinfectants. In some instances, cases can revolve around the different medical procedures linked to infected nonsterile medical equipment [8]. There have been cases of *M. abscessus* outbreaks following the use of contaminated needles and other surgical instruments [2] involving a cohort of “lipotourists” (that is, people who travel abroad for cosmetic surgery for fat removal), which demonstrated severe outbreaks following cosmetic surgery [7].

Increasing commercial propaganda linking beauty with health has led to infections associated with health supplements and surgical procedures [9]. Cosmetics procedures are frequently shown to expose alleged growing organisms and severe abscess infections [1]. Furunculosis due to *M. abscessus* has been observed in patients who have undergone pedicures, acupuncture, liposuction, and other esthetic procedures [9].

The effects of surgical tourism on the healthcare system and individual patients are largely a mystery. Though the exact number is unknown, approximately 80% of patients who undergo plastic surgery in the Dominican Republic are foreign born. We can reasonably estimate that about 11,000 US residents per year undergo plastic surgery in the Dominican Republic. Further evidence of the increasingly high number of people who indulge in surgical tourism was shown when 3–4% of travelers identified “health treatment” as the purpose of their trip in a 2002 survey of US passengers traveling to Central and South America [10]. To effectively treat *M. abscessus* surgical-site infection (SSI), which is very common among patients who have had cosmetic surgery abroad, a multidisciplinary strategy including early aggressive surgical intervention and long-term intravenous antibiotics is essential.

## Conclusion

*Mycobacterium abscessus* infection may occur owing to cosmetic surgery. With the progression of *M. abscessus* infection points examined in our report, we may report on the risks of undergoing surgery in another country. Abscess infection among “lipotourists” from the USA illustrates the potential importance of the internet in identifying and investigating epidemics [7]. For this reason, a wide range of safety rules and health precautions have been implemented. Membrane filtering, hyperchlorination, maintaining consistent pressure gradients, and impregnating certain pipe materials have all been proposed to reduce its concentration in water supply reservoirs [7].

## Data Availability

All data generated or analyzed are included in this article.
